# Disentangling Pectin and Cellulose Nanostructures in Synthetic Plant Cell Walls with Small-Angle Neutron Scattering

**DOI:** 10.1021/acs.biomac.5c02791

**Published:** 2026-04-21

**Authors:** Anna J. Svagan, Olena Kyzyma, Anran Mao, Pramod Sivan, He Li, Agnieszka Ziolkowska, Francisco Vilaplana, Rob Russell, Marité Cárdenas, Elliot P. Gilbert

**Affiliations:** † Department of Fibre and Polymer Technology, Royal Institute of Technology, SE-100 44 Stockholm, Sweden; ‡ Instituto Biofisika (UPV/EHU, CSIC), University of the Basque Country, Barrio Sarriena s/n, 48940 Leioa, Spain; § Department of Chemistry, Division of Glycoscience, Royal Institute of Technology, SE-100 44 Stockholm, Sweden; ∥ Core Facility Electron Microscopy, Umeå University, SE-901 87 Umeå, Sweden; ⊥ National Deuteration Facility, Australian Nuclear Science and Technology Organisation, Locked Bag 2001, Kirrawee DC, Sydney, New South Wales 2232, Australia; # Ikerbasque, Basque Foundation for Science, 48013 Bilbao, Spain; ∇ Biofilms Research Centre for Biointerfaces, Faculty of Health and Society, Malmö University, SE-205 06 Malmö, Sweden; ○ Australian Centre for Neutron Scattering, Australian Nuclear Science and Technology Organisation, Locked Bag 2001, Kirrawee DC, Sydney, New South Wales 2232, Australia

## Abstract

Understanding the plant cell wall architecture is essential for elucidating its biological function and mechanical properties. This study employs a synthetic approach using spherical core–shell capsules with shells composed of deuterated bacterial cellulose (d-BC) and pectin. The shell structure was created via a bottom-up layer-by-layer assembly onto CaCO_3_ templates, followed by characterization through microscopy and scattering techniques. Small-angle X-ray scattering (SAXS) and confocal laser scanning microscopy revealed increased pore sizes in hydrated d-BC/pectin shells compared to those of hydrated wood-derived cellulose nanofiber (CNF)-based shells from a previous study. Using small-angle neutron scattering (SANS) with contrast variation, structural changes of individual wall components under varying salinities (0 or 10 mM NaCl) were analyzed. The presence of NaCl selectively influenced the pectin phase, while the d-BC network retained structural stability, highlighting its robustness as a wall component. This platform provides a useful tool for testing hypotheses and advancing our understanding of cell wall porosity and composition-dependent permeability.

## Graphical abstract

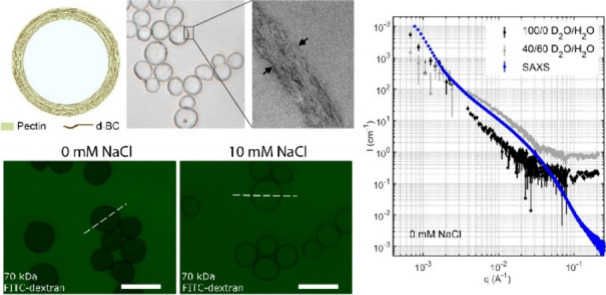

## Introduction

The primary cell wall is a dynamic structure in growing plant cells that provides both the mechanical support and flexibility required for cell expansion. Structurally, the primary cell wall is composed mainly of three classes of polysaccharides, cellulose, hemicellulose, and pectin, along with structural proteins that contribute to wall architecture.^1,2^ Cellulose is present in the form of cellulose microfibrils (CMFs), and the cellulose microfibril network in the wall, governed by cellulose–cellulose contacts, provides mechanical tensile strength and structural integrity to the plant body.^3^ The spatial organization of the cell wall components and their molecular interactions determines the properties of the wall in plant cells.^4^ However, due to the vast diversity of wall polysaccharides and the limited structural understanding of many of these, a complete scientific consensus on the molecular interactions and the diverse roles of primary cell wall components remains elusive.^5^ To unravel the molecular structure–function relationships of cell wall polymers, technological advances and the development of new biomimetic systems are essential.^5^

Simplified cell wall structures can aid in our understanding of the relationship between cell wall morphology and properties because it is possible to limit the number of cell wall constituents to a few.^6–10^ Additionally, they exclude transient phenomena and molecular cues, and most importantly, the often unknown developmental history of wall formation in living plant cells. Recently, it was demonstrated that a synthetic pectin/cellulose nanofiber capsule provides a minimal platform capturing the mechanics of a regenerating plant cell wall.^11^ In another study, the same pectin/cellulose capsules were studied with SAXS.^1^ The walls of these core–shell structures comprised cellulose nanofibers (CNFs) from wood (height of 2.1 nm, AFM) embedded within a matrix of highly esterified pectin. However, despite the utility of SAXS for probing nanoscale features, the technique was limited in its ability to distinguish among the individual wall components. This was due to the similar (X-ray) scattering length densities (SLDs) of crystalline CNFs and the hydrated pectin matrix, resulting in a difference in scattering length density close to zero. While it was therefore not possible to differentiate between these two wall components, the porosity in the wall could be clearly resolved, as the scattering length density difference between water-filled pores and the matrix was one order of magnitude greater compared to that of the wall components.^1^

Small-angle neutron scattering (SANS) is a powerful tool to investigate the nanostructure of plant cell wall materials in their fully hydrated state.^6^ Through contrast variation experiments, SANS enables selective access to scattering data from one component at a time. Here, a selectively deuterated cellulose component was biosynthesized by bacteria (*Gluconacetobacter xylinus*), followed by chemical modification and final mechanical disintegration to yield deuterated bacterial cellulose (d-BC) nanofibers/nanocrystals. Spherical core–shell structures based on d-BC and pectin were then successfully fabricated for the first time, using a bottom-up layer-by-layer (Lbl) assembly strategy. The morphology of these bioinspired structures was characterized using optical and electron microscopy techniques, while small-angle neutron and X-ray scattering were used to assess the nanoscale structural features in the shell. By exploiting the contrast variation capabilities of SANS, changes in the organization of d-BC and pectin were independently evaluated at varying salinity concentrations (0 or 10 mM NaCl). The results are discussed with respect to previous results obtained from SAXS on similar core–shell structures with shells composed of wood-derived cellulose nanofibers (CNFs) and pectin.^1^

## Materials and Methods

### Materials

Pectin (apple, *M*_r_ 30,000–100,000) with a degree of esterification of 70.3%, D_2_O (99.9%), concentrated DCl in D_2_O, 70 kDa FITC-dextrans, Calcofluor White stain (CFW), propidium iodide (PI), cellulase from *Trichoderma* sp. (powder, ≥5000 units/g solid), glycidyltrimethylammonium chloride were purchased from Sigma-Aldrich. CaCO_3_ microparticles were prepared as described in Paulraj et al.^12^ The 6-channel μ-slide VI^0.4^ (ibiTreat) was purchased from Ibidi. The deuterated bacterial cellulose (d-BC) and h-BC (no deuteration) pellicles (bacteria: *Gluconacetobacter xylinus*, ATCC53524) were supplied by ANSTO’s National Deuteration Facility (NDF) in Australia.

### Preparation of Cationic Bacterial Cellulose Nanofibers/Nanocrystals

The d-BC and h-BC pellicles were purified in 1 wt % NaOH at 70 °C for 1 h, followed by excessive washing with 70 °C Milli-Q water. Further purification took place in 8.5 g L^–1^ NaClO_2_ in 0.1 M sodium acetate buffer (pH 4.5–4.6) for 2 h at 70 °C, followed by washing with 0.1 M sodium acetate buffer (pH 4.5–4.6). The purification with 8.5 g L^–1^ NaClO_2_ in 0.1 M sodium acetate buffer was repeated once for the d-BC pellicle. Afterward, the buffer was removed by washing the pellicles with Milli-Q water.

To shorten the bacterial cellulose ribbons, the deuterated BC pellicle was hydrolyzed in a 2.5 M HCl/DCl solution (DCl:HCl = 67:33, molar ratio) for 2 h at 70 °C. The h-BC pellet was hydrolyzed in a 2.5 M HCl solution for 2 h at 70 °C. The products were centrifuged (16k rpm, SORVALL RC 5B PLUS centrifuge, Fiberlite F21-8 × 50y rotor) for 5 min, and the supernatants was discarded. The sediments were diluted back to the same volume with Milli-Q water and dialyzed (cellulose membrane tube, MWCO = 12–14 kDa) against Milli-Q water until the surrounding media reached a stable pH. d-BC and h-BC samples were taken out for zeta potential, D/H ratio analysis, and FTIR analysis.

#### Chemical Modification with Glycidyltrimethylammonium Chloride

NaOH dissolved in 65:35 (mol %) D_2_O:H_2_O was added to the remaining d-BC sample, and the suspension (with 4.9 wt % NaOH) was left standing overnight at RT. The suspension was concentrated by centrifugation to ca. 2.5 wt % cellulose (dry content of d-BC), and glycidyltrimethylammonium chloride was added (10 times the dry weight of cellulose), and the reaction proceeded at 65 °C for 8 h. The modified d-BC suspension was centrifuged (5 min, 16 krpm, SORVALL RC 5B PLUS centrifuge, Fiberlite F21-8 × 50y rotor), and the supernatant was discarded, and Milli-Q water was added to the same volume as prior to centrifugation. The centrifugation step was repeated once. Afterward, the product was dialyzed (cellulose membrane tube, MWCO = 12–14 kDa) against Milli-Q water until the pH of the surrounding medium was stable (4 days). The final d-BC suspension had a pH of 7.2 and a dry content of 0.36 wt % (measured with thermogravimetric analysis). The surface charge was 0.11 ± 0.01 mmol g^–1^, obtained by measurements (*n* = 2) on the d-BC suspension (ca. 5 mL) using conductometric titration (AgNO_3_, 5 mM).^13^

Prior to Lbl assembly, the d-BC suspension was diluted to 0.05 wt %, and the pH was adjusted to 7.5 ± 0.2, followed by tip sonication (1/2-in. tip, 80% amplitude, 1 min, 25 mL, Vibra-Cell VCX 750, Sonics & Materials) and finally centrifugation for 5 min (16k rpm, SORVALL RC 5B PLUS centrifuge, Fiberlite F21-8 × 50y rotor). The supernatant was collected (the sediment was discarded), and NaCl was added to a concentration of 100 mM NaCl. When preparing the AFM sample, no NaCl was added; instead, the sample was further diluted to 0.005 wt %.

### Preparation of Layer-by-Layer d-BC/Pectin Core–Shell Structures on Top of CaCO_3_ Particles

The layer-by-layer assembly of pectin and d-BC on CaCO_3_ template particles was performed as described in detail previously,^1,12,14^ with the modification that 1.6 mL (instead of 2 mL) of d-BC (0.05 wt %, pH 7.5 ± 0.2) and pectin (0.1 wt %, pH 7.5 ± 0.2) was used in the Lbl assembly. All suspensions contained 100 mM NaCl. In the end of the assembly protocol, the multilayer-coated CaCO_3_ particles were washed thoroughly with water to remove NaCl, and the d-BC/pectin-coated CaCO_3_ particles were dried at 50 °C. Core removal was performed using a 50 mM citric acid solution, followed by extensive washing with Milli-Q water, as described previously.^1^

### Characterization

#### Fourier-Transform Infrared Spectroscopy (FTIR)

Spectra were obtained with a PerkinElmer Spectrum 2000 FTIR equipped with an MKII Golden Gate single-reflection ATR system. Measurements were conducted from 600 to 4000 cm^–1^ with a 4.0 cm^–1^ resolution. Prior measurements, droplets of the d-BC suspension (d-BC without and with cationic surface groups) were dried on glass slides at 40 °C. The dry sample was peeled off from the glass using a scalpel blade.

#### D/H Ratio Analysis of Bacterial Cellulose

An amount of 1–2 mL of d-BC or nondeuterated BC (h-BC) (both purified and hydrolyzed, no cationic surface groups, dry content of 0.1 wt %) was enzymatically degraded with cellulase (0.2 mg cellulase/mg cellulose) in 50 mM citrate buffer (pH 4.8). The blank sample contained 50 mM citrate buffer and 0.2 mg cellulase mL^–1^. All samples were kept at 40 °C for 24 h. The products were filtered through a 0.45 μm PTFE syringe filter. The composition was analyzed with liquid chromatography-electron spray ionization mass spectrometry. Samples were briefly passed through a ZORBAX Eclipse Plus C18 column 1.8 μm (2.1 × 50 mm, Agilent Technologies, Santa Clara, CA) at a flow rate of 0.1 mL min^–1^ and a gradient of increasing acetonitrile content (10–30%) over 6 min. Mass spectrometric (MS) analysis was performed by electrospray ionization mass spectrometry (ESI-MS) using a Synapt HDMS mass spectrometer (Waters, USA) in positive-ion mode. The capillary and cone voltages were set to 3 kV and 70 V, respectively. The degradation products were detected as [M + Na^+^] adducts.

#### Chemical Characterization

Following the sulfuric acid hydrolysis protocol, pectin, Lbl capsule, and d-BC materials (ca. 1 mg of dry material in glass tubes, duplicate samples) were mixed with 125 μL of 72% (w/w) H_2_SO_4_ and subsequently hydrolyzed.^15^ The samples were incubated at room temperature for 3 h. An amount of 1375 μL of Milli-Q water was added, and the hydrolysis was continued at 100 °C for an additional 3 h. Samples were cooled to room temperature.

Following the trifluoroacetic acid (TFA) hydrolysis protocol, 1 mg of dry sample (pectin or Lbl capsule material) was accurately weighted in duplicate into glass tubes.^15^ Each tube was then treated with 1 mL of 2 M TFA and incubated at 120 °C for 3 h. The samples were cooled to room temperature and dried by evaporation in a ventilated fume hood. The dried residues were dissolved in 1 mL of Milli-Q water.

All hydrolyzed samples were diluted 1:10 by combining 100 μL of hydrolysate with 10 μL of 1 mg/mL of inositol (used as an internal standard) and 890 μL of Milli-Q water in 1.5 mL Eppendorf tubes. The diluted solutions were filtered through a Chromacol 17-SF-02­(N) syringe filter (17 mm diameter, 0.2 μm nylon membrane) prior to chromatographic analysis. Monosaccharide composition was determined using high-performance anion-exchange chromatography with pulsed amperometric detection (HPAEC-PAD), performed on a Dionex ICS-6000 system equipped with a CarboPac PA20 analytical column. The column was maintained at 30 °C with a flow rate of 0.4 mL min^–1^. Appropriate elution gradients were applied to separate both neutral and acidic sugars.^16^ Quantification was performed using monosaccharide standards, including fucose, rhamnose, arabinose, xylose, glucose, galactose, mannose, galacturonic acid, and glucuronic acid. The chemical composition of the neat pectin and pectin present in the Lbl capsules, deduced from TFA hydrolysis, is reported in Table S1 in the SI. The d-BC content (wt %) of the Lbl capsule was quantified via the sulfuric acid protocol, using the measured glucose concentration (mg/g sample) for the d-BC, pectin, and Lbl capsule samples in the calculations. The pectin content in the Lbl capsule was calculated as the remaining fraction after subtracting the measured d-BC content.

#### Zeta Potential Measurements

Measurements were performed using a Zetasizer Nano ZS ZEN3600 (Malvern Instruments Ltd., U.K.). Suspensions with d-BC without and with cationic surface groups (devoid of NaCl) were diluted to 0.05–0.08 wt % prior to measurements. Pectin solutions (0.1 wt %) were adjusted to pH 7.5 ± 0.2 and filtered through a 0.8 μm syringe filter. The Smoluchowski approximation was used.

#### Atomic Force Microscopy (AFM)

AFM was performed with a MultiMode 8 AFM (Bruker, Santa Barbara, CA) and ScanAsyst-Air Cantilevers (PeakForce QNM mode). 20 μL of the cationic d-BC suspension (0.005 wt % in Milli-Q) was dropped onto freshly cleaved mica, immediately followed by Milli-Q water rinsing and drying (nitrogen gas). Gwyddion software (version 2.63) was used to evaluate the height, length, and aspect ratios (*n* = 110–120) of the d-BC.

#### Light Microscopy

Images were obtained using an Axio Vert.A1 light microscope (Carl Zeiss, Germany) equipped with cross-polarizers.

#### Transmission Electron Microscopy (TEM)

Micrographs were acquired using a Thermo Scientific Talos L120C transmission electron microscope operated at an accelerating voltage of 120 kV. The core-removed Lbl core–shell structures, suspended in water, were stained with 0.05% malachite green and then cross-linked with 2.5 wt % glutaraldehyde for 2 h. Following cross-linking, samples were gradually dehydrated in absolute ethanol and embedded in epoxy resin (Epoxy Embedding Medium Kit, Sigma-Aldrich) using polyethylene molds (BEEM Capsules 00). The resin was polymerized in a stepwise manner at 45 °C for 12 h, followed by 60 °C for 24 h. Ultrathin sections (≈70 nm) were prepared using a Leica EM FC7 ultramicrotome. Prior to imaging, the sections were stained with 5 wt % aqueous uranyl acetate for 45 min.

#### Scanning Electron Microscopy (SEM)

Micrographs were obtained with a Hitachi SEM S-4800 instrument (acceleration voltage of 1 kV). The Lbl-coated CaCO_3_ particles in water were deposited onto Si wafers and allowed to dry, facilitating adhesion to the substrate. The CaCO_3_ cores were then selectively removed by treatment with 50 mM citric acid solution. Following core dissolution, the samples were rinsed thoroughly with Milli-Q water, dried, and subsequently imaged.

#### Confocal Laser Scanning Microscopy (CLSM)

CLSM was performed using an LSM 780 (Carl Zeiss, Germany). Excitation wavelengths were 488 nm (FITC-dextran), 405 nm (CFW stain), and 514 nm (PI). Permeability experiments were performed as described in detail previously,^1,17^ by adding 70 kDa FITC-dextrans (in 0 or 10 mM NaCl) to CaCO_3_ core-removed core–shell structures in Ibidi channels, with the medium in the channel being either 0 or 10 mM NaCl.

#### Synchrotron SAXS and WAXS

Measurements were carried out at the CoSAXS beamline at Max IV Laboratory (Lund, Sweden) using an X-ray wavelength of 1 Å. The beam was focused to approximately 150 × 150 μm (width × height) at a position 3.5 m upstream of the sample position. Samples were loaded into borosilicate glass capillaries (wall thickness: 0.01 mm, outer diameter: 1.5 mm) and mounted in a temperature-controlled multicapillary sampled holder maintained at 21.2 ± 0.3 °C. SAXS and WAXS data were collected simultaneously. SAXS patterns were recorded using an EIGER2 4 M detector (Dectris AG) positioned at two sample–detector distances: 3.46 and 14.10 m. The WAXS data were acquired with a custom “L-shaped” PILATUS3 × 2 M detector (Dectris AG) at a fixed distance of 0.46 m. The beam center and the 3.46 m SAXS and WAXS sample–detector distances were calibrated using silver behenate as a standard, mounted in a capillary within the sample holder. For the 14.1 m configuration, data calibration was performed from the pixel position of an attenuated direct beam on the SAXS detector and the additional distance the SAXS carriage was moved from the 3.46 m position. Measurements were conducted on core–shell samples dispersed in Milli-Q water or on the solvent (Milli-Q) background alone. Each capillary was vertically scanned through the beam in 19 discrete steps, with 50 ms exposures per position. Given the 150 μm vertical beam height, each exposure probed a fresh section of the sample. The two-dimensional SAXS and WAXS patterns were automatically reduced to 1D scattering patterns using the Azint software suite provided by MAX IV.^18^ Very large-scale diffraction investigations were enabled by a matrix-multiplication-facilitated radial and azimuthal integration algorithm: MatFRAIA.^18^ Transmission normalization was performed using incident and transmitted X-ray intensities measured by photodiodes placed before the sample and at the beam stop in front of the SAXS detector. These incident and transmitted X-ray intensities were corrected with respect to the detector dark current (“Black current”). The 19 individual 1D scattering patterns for each sample were averaged, solvent background was subtracted, and data from different sample–detector distances were merged based on overlapping *q*-ranges using custom scripts in MATLAB.

### Neutron Scattering (SANS)

SANS experiments were conducted on the QUOKKA instrument at the OPAL reactor at ANSTO.^19^ Four instrument configurations were used to cover the full *q*-range. Configurations with sample–detector distances of 1.3, 8, and 20 m used a neutron wavelength of 5.0 Å, while a further 20 m focusing lens configuration used a wavelength of 8.1 Å, all with a source aperture of 50 mm, a sample aperture diameter of 12.5 mm, and a wavelength resolution, Δλ/λ, of 0.10, except for the lens configuration, where a source aperture of 30 mm was employed. Dispersions of d-BC (6.6 mg/mL) and pectin (10 mg/mL) were prepared in a series of D_2_O/H_2_O mixtures to identify the optimal contrast-matching conditions (Figure S1, Supporting Information). The d-BC mixtures were prepared by combining stock dispersions of d-BC in water and d-BC in D_2_O in defined volumetric ratios. Pectin pellets were dissolved directly in the selected D_2_O/H_2_O solvent mixtures. These pectin pellets were prepared by dissolving pectin in Milli-Q water (0.1 wt %), adjusting the pH to 7.5 ± 0.2, followed by filtration through a 0.8 μm syringe filter. The filtered solution was then dried at 40 °C to obtain the pectin pellets used in subsequent contrast-matching experiments. The resulting data were subsequently used to define solvent ratios. Measurements were thus conducted on d-BC/pectin Lbl core–shell Lbl samples dispersed in different D_2_O/H_2_O media (100/0 and 40/60) or on the solvent backgrounds alone. The Lbl sample concentration in 100/0 (D_2_O/H_2_O) was ca. 1 vol %, whereas the concentration was ca. 0.8 vol % for Lbl samples in 40/60. The samples were loaded into 1 mm path length cylindrical quartz glass cuvettes (Hellma, 280 μL, “banjo cells”, outer width: 22 mm). The cuvettes were tumbled during measurements to avoid sedimentation of the core–shell Lbl samples. Following initial measurements, a small volume of concentrated NaCl solution (1 M) in either 100% D_2_O or 40/60 D_2_O/H_2_O was injected into each cuvette to reach a final NaCl concentration of 10 mM. The same samples were then remeasured under identical conditions. All data were reduced using NIST Centre for Neutron Research SANS reduction macros,^20^ modified for the QUOKKA instrument, using the Igor software package (Wavemetrics, Lake Oswego, OR), and corrected for solvent background, transmission, and detector sensitivity. The data were transformed onto an absolute scale using attenuated direct beam transmission measurements.

### SAS Data Analysis and Interpretation

The scattering data were analyzed using SASview (version 5.06, https://www.sasview.org) with the DREAM fit optimizer. The SAXS data were fitted with a core–shell sphere model (for the low-*q* region) and two cylinder models (pores) at higher *q*. The core radius and distribution were allowed to vary around values obtained for the Lbl capsules from light microscopy. The resulting best-fit values were then used as fixed inputs in the core–shell sphere model during analysis of the SANS data. Similarly, the optimized shell thickness parameter obtained from the SAXS core–shell sphere fits was fixed for subsequent fitting of SANS data collected in 100/0 D_2_O/H_2_O (at 0 mM NaCl only). SANS intensity curves for the 100/0 D_2_O/H_2_O samples (0 mM and 10 mM NaCl) and for the 40/60 samples in 10 mM NaCl were fitted using a combined core–shell sphere and Debye–Anderson–Brumberger (DAB) model (Table S2, Supporting Information). For the core–shell sphere component, the scale factor was fixed to the concentration of the Lbl capsules, i.e., 0.01 for the 100/0 samples and 0.008 for the 40/60 sample (the overall scale was set to 1). The remaining free parameters for the DAB model and the core–shell sphere model: shell SLDs and shell thickness distributions (fixed for the 100/0 in 10 mM NaCl sample), and, for the 100/0 (at 10 mM NaCl) and 40/60 samples, also the shell thickness, were fitted; the best-fit values are summarized in Table S2.

## Results and Discussion

### Morphology of Cationic Bacterial Cellulose Nanofibers/Nanocrystals (BC)

Compared to wood-derived cellulose nanofibers, bacterial cellulose (BC) is a pure cellulose material, lacking associated cell wall matrix components such as lignin, hemicellulose, and pectin.^21^ This intrinsic purity simplifies downstream processing because BC requires only mild purification treatment with dilute NaOH at elevated temperatures to remove residual cellular debris formed on the surface of the bacterial cellulose ribbons during microbial secretion.^10^ Furthermore, it can readily be deuterated by culturing cellulose-producing bacteria on a deuterated carbon source. A fraction of the protium (the most common isotope of hydrogen) in the glucose units will thereby be replaced with deuterium, which increases the neutron scattering length density (SLD) of the cellulose, enhancing contrast in SANS experiments. Structurally, bacterial cellulose nanoribbons are similar to cellulose microfibrils found in plant cell walls, although notable differences exist.^22^ Native bacterial cellulose ribbons have widths of ∼ 30–50 nm and lengths extending several micrometers^22,23^ ; they result from aggregation of several elementary fibrils (≈1.5–6 nm in thickness)^22^ into microfibrils, followed by subsequent bundling of microfibrils and finally aggregation of these bundles into ribbons.^23^ In contrast, cellulose microfibrils in plant cell walls have dimensions (width and thickness) closer to the thickness of the elementary fibrils of BC.^1,24,25^ In this study, the deuterated BC ribbons were subjected to controlled acid hydrolysis and chemical modification, followed by mechanical disintegration, yielding a mixture of finer nanostructures. Atomic force microscopy (AFM) revealed that the resulting cationically charged d-BC nanofibers had an average thickness of 6.1 ± 2.3 nm and an average length of 970 ± 720 nm, corresponding to an average aspect ratio (*L*/*d*) of approximately 180 (Figure 1a; see histograms in Figure S2, Supporting Information). BC nanocrystals prepared through hydrolysis of the cellulose ribbons have a thickness of ca. 5–10 nm and lengths below 1000 nm.^26^ Thus, our d-BC particles are best described as a population consisting of both cellulose nanofibers and nanocrystals.

**Figure 1:**
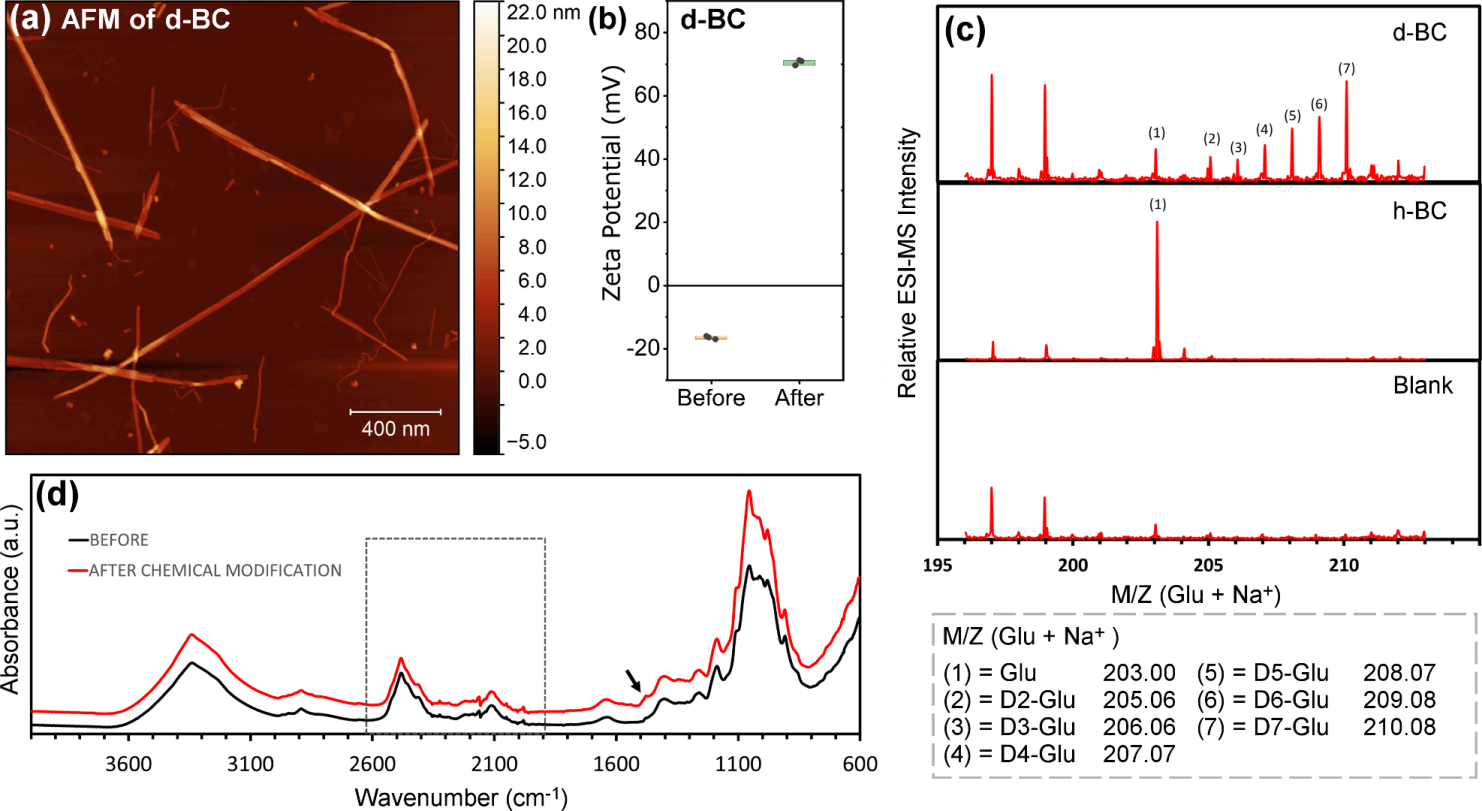
Characterization of deuterated bacterial cellulose (d-BC), (a) atomic force microscopy (AFM) image of cationic deuterated bacterial cellulose nanocrystals/nanofibers. Scale bar: 400 nm. (b) Zeta potential (*n* = 3) of d-BC before (−17 ± 0.5 mV) and after chemical modification (+71 ± 0.9 mV, mean ± s.d.), confirming the introduction of cationic surface groups. (c) Deuterium/hydrogen (D/H) composition of d-BC, native BC (h-BC, 100% H), and a blank sample (buffer medium + cellulase). All three samples contained trace amounts of cellulase. (d) ATR-FTIR spectra of d-BC before and after chemical modification; spectra are vertically offset for clarity. The arrow points to a shoulder at 1480 cm^–1^. The dashed-line box: the O–D stretching band at 2480 cm^–1^ and C–D bond stretching at 2160 cm^–1^.

The presence of cationic surface groups on the d-BC, introduced via chemical modification, was confirmed by ζ-potential measurements (Figure 1b). Successful deuterium labeling of the d-BC was verified by analyzing the enzymatic degradation products (glucose) using mass spectrometry (Figure 1c) and d-BC by FTIR spectroscopy (Figure 1d). The resulting deuterated bacterial cellulose (d-BC) and the D/H composition of d-BC, are presented in Figure 1c. The D/H composition of the d-BC was determined from the mass spectra of the hydrolysis products, with samples analyzed prior to chemical modification. For comparison, glucose fragments from nondeuterated bacterial cellulose (h-BC) and a blank sample containing only buffer and enzymes were also included in the analysis. This allowed for the identification and exclusion of background molecules originating from the enzyme mixture or buffer. As shown in Figure 1c, the degradation products of d-BC contained between two and seven deuterium atoms per glucose unit. The most abundant species corresponded to glucose molecules in which all seven exchangeable hydrogens were replaced with deuterium. FTIR analysis further supported partial deuterium labeling: a broad O–H stretching band at 3340 cm^–1^ and a characteristic O–D stretching band at 2480 cm^–1^ were observed, along with C–D bond stretching at 2160 cm^–1^ (Figure 1d), consistent with previously reported spectra.^9,27^ These bands remained comparable in intensity and position before (black line) and after (red line) chemical modification, indicating that deuteration was retained throughout the modification process. The chemical functionalization introduced cationic quaternary ammonium groups onto the d-BC surface, evidenced by the new band observed as a shoulder at 1480 cm^–1^ (marked by an arrow in Figure 1d), which has previously been assigned to bending vibrations in methyl groups and the scissoring vibration of the −CH_2_– group attached to the N atom in the quaternary ammonium moiety.^28,29^

### Morphology and Barrier Properties of the Lbl Capsules

Alternating layers of d-BC nanocrystals/nanofibers and pectin were sequentially adsorbed onto sacrificial spherical CaCO_3_ templates to form a multilayer shell structure.^1,12,14^ Such shell assemblies are also known as polyelectrolyte multilayers (PEMUs), and in the present study, the shell consisted of a total of six bilayers; one bilayer consists of one adsorbed pectin layer and one adsorbed d-BC layer. However, we note that the multilayers are not stratified into discrete pectin and CNF layers but are instead, to some extent, dispersed and interpenetrating, as depicted in Figure 2c.^30^ The CaCO_3_ core was later removed using citric acid, producing the liquid-core Lbl-shell capsules (Figure 2a). Similar to polyelectrolyte complexes (PECs), the PEMUs form through charge-pairing interactions between oppositely charged polycations (here d-BC, zeta potential ξ = +71 mV, Figure 1b) and polyanions (here pectin, zeta potential ξ = −46 ± 1 mV, mean ± s.d., *n* = 3). The major driving force for the oppositely charged polyion association is the entropic release of counterions and water.^31–33^ In addition to electrostatic interactions, nanofiber–nanofiber interactions, including van der Waals interactions and hydrogen bonding, are expected to contribute to the intermolecular forces present in the shell.

**Figure 2:**
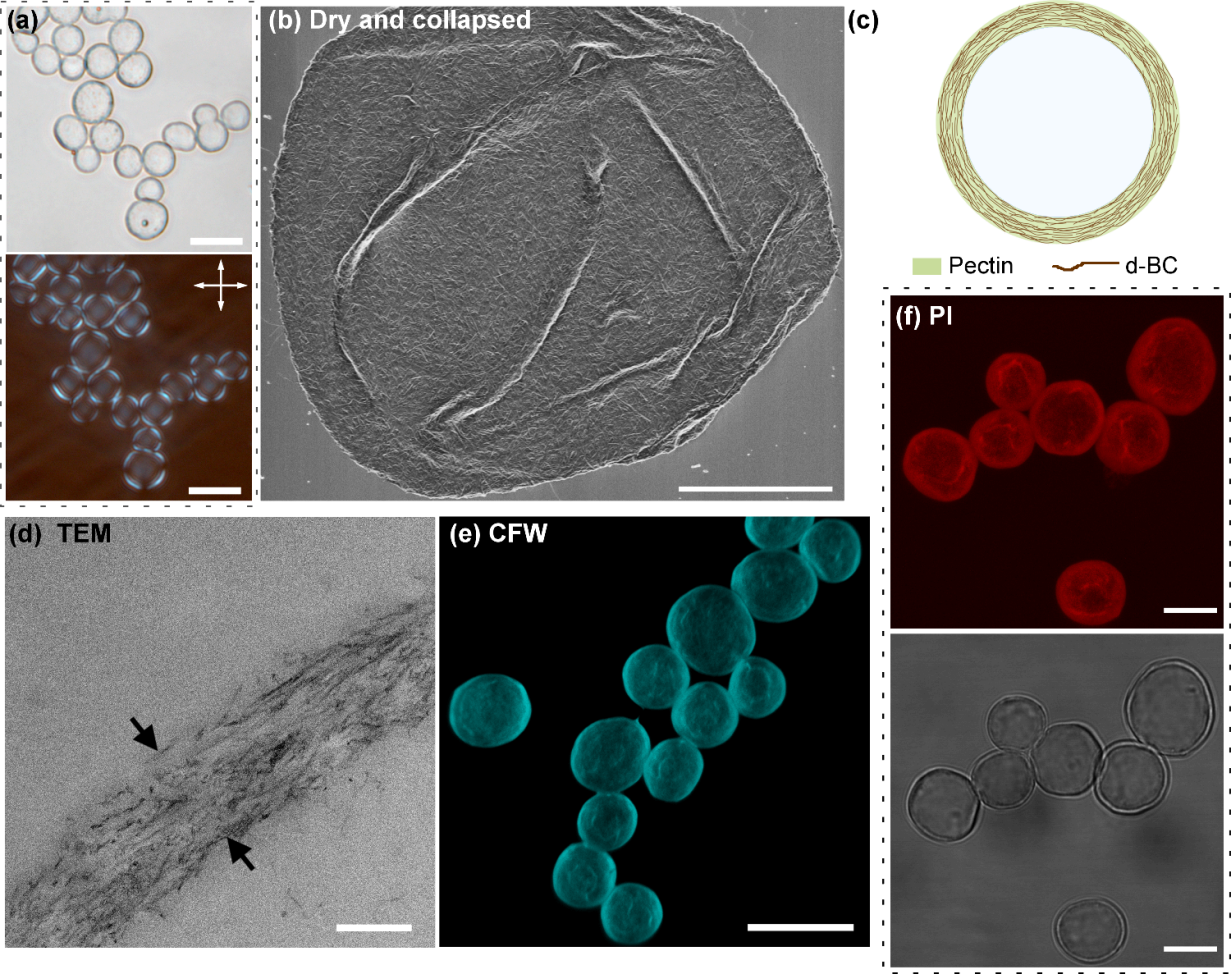
Morphology characterization of spherical core–shell Lbl structures with shells consisting of six bilayers. (a) Bright-field and polarized optical microscopy (POM) images of core-removed Lbl shells. (b) SEM images of a dried and collapsed spherical core–shell Lbl d-BC/pectin structure following core removal. (c) Simplified schematic illustrating the cross-section of a spherical core–shell structure. (d) TEM image showing a cross-section of the Lbl shell. CLSM images of spherical core–shell Lbl d-BC/pectin structures stained with (e) calcofluor white stain (CFW) and (f) propidium iodide (PI). A transmission image is included in (f). Scale bars: 20 μm (a, e), 5 μm (b), 200 nm (d), and 10 μm (f).

Due to the differences between bacterial cellulose and, for example, wood-derived cellulose nanofibers, in terms of fiber length, thickness, and crystalline domain length, there may be geometric limitations to how deuterated bacterial cellulose (d-BC) assembles around curved surfaces. However, in the present study, these constraints were effectively circumvented by employing relatively large calcium carbonate (CaCO_3_) templates (>10 μm in diameter). Notably, native bacterial cellulose nanocrystals have previously been reported to stabilize spherical oil droplets with diameters as small as 5 μm,^34^ demonstrating the flexibility of bacterial cellulose in adapting to spherical geometries under appropriate conditions. The diameter of the final Lbl capsules was 12.7 ± 2.1 μm (*n* = 600), as determined by optical microscopy (Figure S3, Supporting Information). Bright-field and polarized optical microscopy (POM) images of the Lbl capsules are shown in Figure 2a. In all constructs, the outermost layer of the Lbl shell consisted of deuterated bacterial cellulose. The birefringent Maltese cross patterns observed in the POM images confirm an anisotropic, layered architecture of the capsule shells.^1^ In the dry state, SEM (Figure 2b) revealed apparent surface pores in the collapsed Lbl shells. The average pore diameter was 89 ± 53 nm, as quantified in Figure S4 (Supporting Information). These pores are expected to decrease in size in the hydrated state due to swelling of the pectin matrix, which governs pore dimensions.^1^ Transmission electron microscopy (TEM) of cross-sectioned capsules showed a clearly defined multilayer shell structure with an average shell thickness of 280 ± 60 nm (Figures 2d and S5, Supporting Information). CLSM microscopy, following staining with calcofluor white (CFW) and propidium iodide (PI), confirmed the presence of cellulose and pectin, respectively, within the Lbl shell (Figure 2e,f). Compared to previously reported Lbl structures assembled from wood-derived cellulose nanofibers (CNFs) and pectin,^1^ the d-BC/pectin capsules exhibited thinner shells and larger surface pores in the dry state.

The assembled spherical core–shell structures were further exposed to a 70 kDa FITC-dextran (average hydrodynamic diameter ca. 12 nm, Figure S6) in Milli-Q water to evaluate any differences in barrier properties, and indirectly morphological differences, between the d-BC/pectin shells and the previously reported wood-CNFs/pectin shells.^1^ The fluorescent dextran entered the interior of the d-BC/pectin capsules, and a higher normalized fluorescence intensity (*I*/*I*_0_) was observed within the capsules compared to previous CNF/pectin-based core–shell structures (Figure 3a,c).^1^ In nonsaline conditions (pure water, Figure 3a,c), the complexation between pectin and d-BCs remains intact, preserving the integrity of the PEMU.^32,35–40^ Consequently, a larger pore diameter within the percolation network of pores in the d-BC/pectin shell, compared to previously reported wood-CNF/pectin shells, is required to allow larger molecules in the 70 kDa dextran population to permeate through the shell. The difference in dextran permeability between the present and previous wood-CNF-based structures may be attributed to several factors, including geometrical restrictions imposed by the long and rigid d-BC nanorods, leading to a different structural organization in the shell. Such geometrical restrictions could influence the packing density of the d-BC in the shell. Second, reduced surface charge density on the d-BC likely limits the extent of pectin adsorption. The cationic surface charge of the d-BC was measured at 0.11 ± 0.01 mmol g^–1^, whereas the CNFs used previously exhibited a cationic surface charge an order of magnitude higher (1.17 mmol g^–1^).^1^ Changing the surface charge density on the d-BC is expected to alter the weight ratio of pectin and d-BC in the shell. Pectin has previously been invoked as the determinant of the cell wall porosity,^41,42^ and a lower pectin content, due to less adsorption in the current capsule shells, may account for the increased dextran permeability in aqueous (nonsaline) conditions. The thinner shells of the present structures (Figure 2d) provided further evidence of less material deposition during the layer-by-layer buildup.

**Figure 3:**
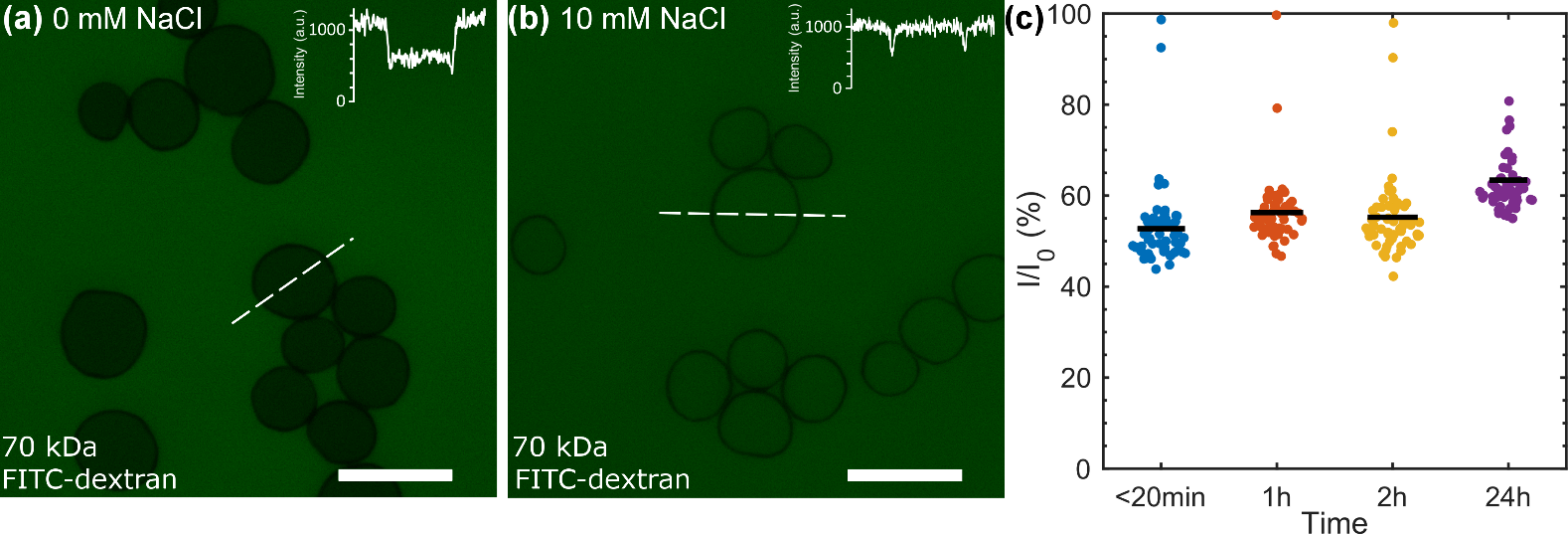
CLSM analysis of core–shell structure permeability. d-BC/pectin core–shell structures exposed to 1 mg mL^–1^ 70 kDa FITC-dextrans present in (a) water for 2 h and (b) after 19 min of incubation in 10 mM NaCl. (c) Fluorescent intensity ratio (*I*/*I*_0_), where *I* is the internal fluorescence intensity and *I*_0_ is the external intensity, measured over time in water (*n* = 60). Scale bars: 20 μm.

When the capsules were exposed to 10 mM NaCl, the FITC-dextran entered the capsules rapidly (within minutes), and the internal fluorescence intensity became comparable to the external medium (Figure 3b). This salt-enhanced dextran permeability response is consistent with that observed in wood-CNF/pectin Lbl capsules under similar ionic strength conditions.^1^ ^,^ ^12^ Adding salt, also referred to as “doping”, breaks pairing interactions between polycations and polyions, and additional water usually accompanies doping. These effects reversibly plasticize the polymers (“saloplasticity”).^32^ A previous study showed that a PEMU can swell upon exposure to solutions containing salt,^43^ and in other polyelectrolyte complexation studies, salt resistance has been used as a measure of the strength of the polyelectrolyte complex.^32,35–40^ However, as shown previously for wood-CNF/pectin Lbl, the salt effect is reversible, and by washing away the NaCl, the barrier properties of the shell can be restored.^12^

### Structural Characterization with Small-Angle Neutron Scattering

To investigate the individual nanostructures of the d-BC and the pectin within the shell, and the extent to how these change in the presence of salt (10 mM NaCl), SANS measurements were conducted. The contrast variation method was performed using different D_2_O/H_2_O ratios to selectively match out one of the components, either pectin or cellulose, in the shell. At a D_2_O/H_2_O ratio of 40/60, pectin is matched out and thus rendered “invisible” in the scattering profile, whereas at 100% D_2_O the d-BC was matched out (see Figure S1 in the SI). Figure 4 presents the SANS results at the two solvent contrast conditions (40/60 and 100/0 D_2_O/H_2_O) and at two NaCl concentrations (0 and 10 mM). In all cases, scattering at low *q* (≈0.0007–0.005 Å^–1^) is predictably dominated by the spherical core–shell structure (see fits in insets in Figure 4a,b). However, we note that the lens configuration data at low *q*, appropriate to investigate these larger-sized objects, inevitably suffer from poorer statistics due to the reduced beam intensity at the longer wavelengths used. Also, we note we have limited sensitivity to the dimensions of the larger core–shell sphere lengths because the lowest *q* value in the experiment is 0.0007 Å^–1^; hence, the shell thicknesses reported in Table S2 should be treated as an approximation only. While ultra-SANS measurements may assist, it is noted that given the mean diameter and size distribution of the capsules, their dimensions would still be outside the typical range of this technique.

**Figure 4:**
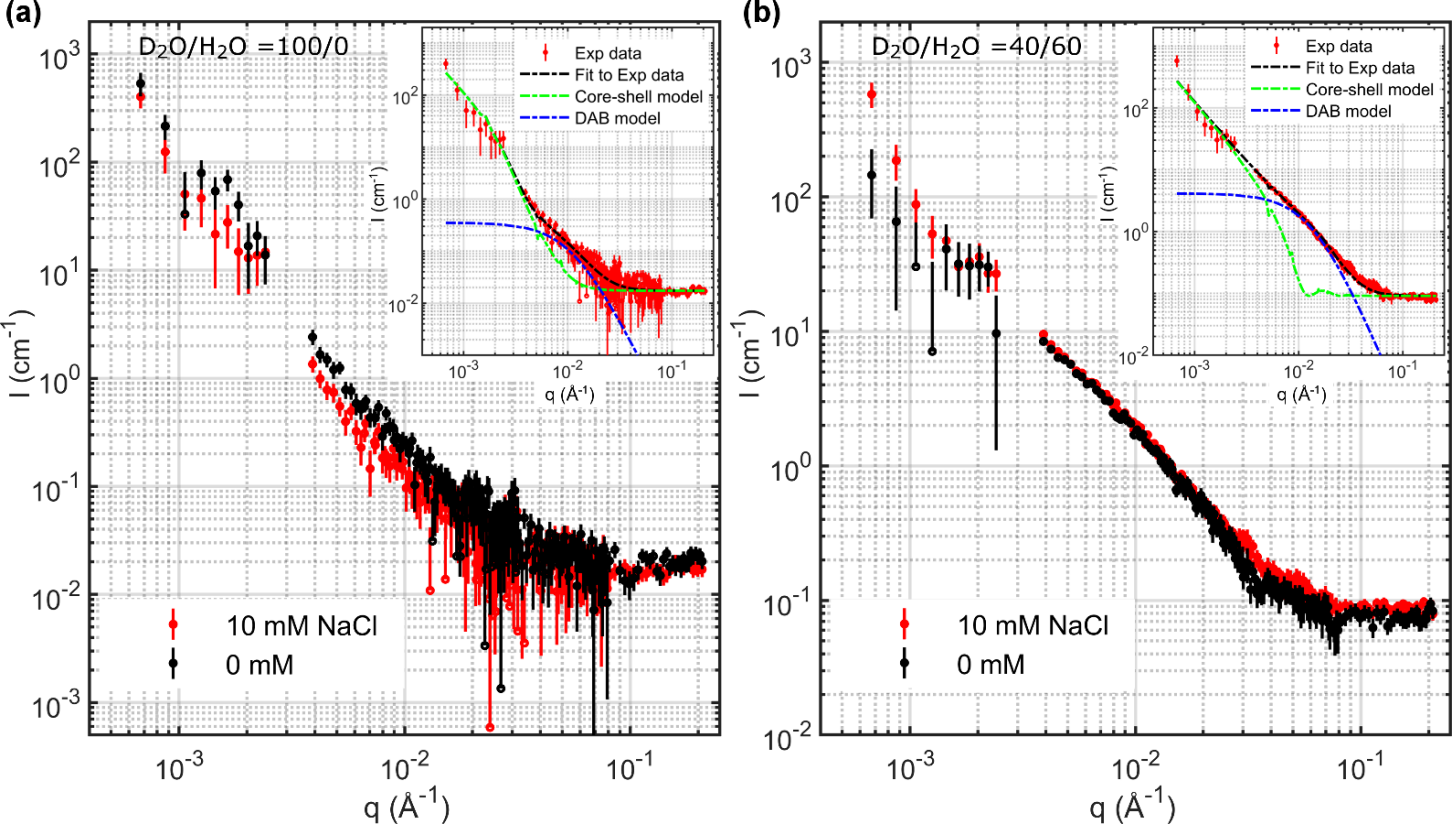
SANS data for Lbl core–shell structures in (a) 100% D_2_O and (b) 40/60 D_2_O/H_2_O mixtures, each at two NaCl concentrations (0 and 10 mM NaCl). In (a), pectin is visible (cellulose contrast-matched), while in (b), cellulose is visible (pectin contrast-matched). Inset in (a) and (b) experimental data overlaid with model fits (dotted black line). The individual contributions of the core–shell sphere model (green) and the Debye–Anderson–Brumberger (DAB) model (blue) are shown separately.

The high *q* regions (≈0.005–0.1 Å^–1^) were further analyzed using the Debye–Anderson–Brumberger (DAB) model (see insets in Figures 4 and S7 in the SI).^44^ This model is suitable for describing two-phase disordered systems and is given by

$$I(q)=\frac{A\cdot L^{3}}{(1+q^{2}L^{2}{)}^{2}}$$ 1

where *L* is the correlation length, which is a measure of the average spacing between regions of the two phases, as illustrated in Figure 5. At 100% D_2_O, the primary phase includes the hydrated pectin, and the second phase includes the pore fraction, containing D_2_O, and the contrast-matched hydrated d-BC. The scale *A* is defined as

$$A=8\pi \cdot \phi (1-\phi )\Delta \rho^{2}$$ 2

where ϕ is the volume fraction of the second phase (at 100% D_2_O, the hydrated pores and contrast-matched d-BC), and Δρ is the scattering length difference between the two phases. At 40% D_2_O, the primary phase will be the hydrated d-BC, whereas the second phase will be the liquid-filled pores and the contrast-matched hydrated pectin.

**Figure 5:**
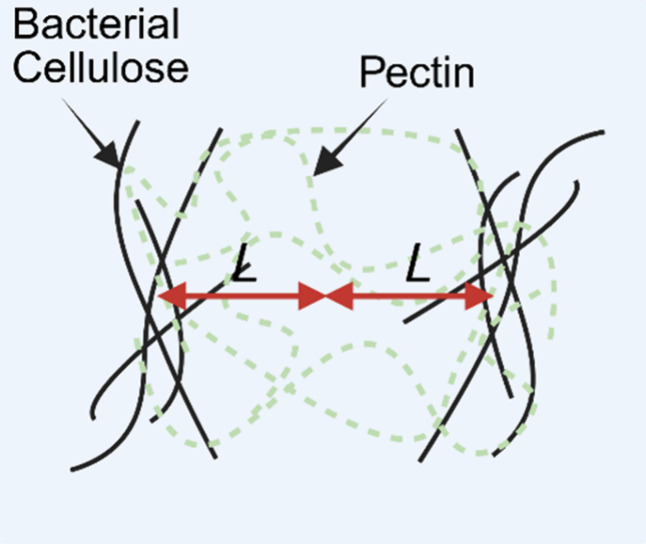
Simplified schematic illustrating the correlation length, *L*, as defined in SasView (SasView 6.1.2 documentation). In a two-phase system, the correlation length is a measure of the average spacing between regions of Phase 1 and Phase 2 (cellulose and pectin). Figure created with BioRender.

Upon addition of NaCl at 100% D_2_O, a small decrease in the scattering slope was observed, suggesting minor structural changes in the shell; compare the black and red scattering curves in Figure 4a at *q* ≈ 0.004–0.01 Å^–1^. The optimized fitting parameters are summarized in Table S2 in the Supporting Information. A small decrease, on the order of ca. 1 nm (within error, see Table S2), in the correlation length, *L*, was observed, that is from 99.8 Å (0 mM NaCl) to 90.1 Å (10 mM NaCl). This minor decrease in correlation length suggests a change to slightly smaller and more finely dispersed heterogeneities.^44^ A change was additionally found in the scale factor, *A*, which decreased by 40%, from 8.0 × 10^–7^ Å^–3^ cm^–1^ (0 mM NaCl) to 4.8 × 10^–7^ Å^–3^ cm^–1^ (10 mM NaCl). ϕ­(1 – ϕ) is a downward-opening quadratic function, and when ϕ > 0.5, a small increase in ϕ gives a lower value for ϕ­(1 – ϕ) and thus would lead to a smaller *A*-value (if Δρ were constant). We attribute the reduction in the *A*-value here primarily to a decrease in the SLD contrast Δρ, rather than to a significant change in the volume fraction of the second phase (ϕ).

From sugar analysis, we see that the dry weight ratio between pectin and d-BC is 17.5:82.5 (wt %:wt %), making d-BC the most abundant matrix component in the dry shell. Hence, if we assume that cellulose and pectin are hydrated to the same extent,^1^ it is reasonable to assume that the volume fraction of the second phase (pores and d-BC) is larger than 0.5, but only the pores in the wall will contribute to changing ϕ because the volume of contrast-matched d-BC nanofibers/nanocrystals is not expected to change with salinity (d-BC will not swell). However, we note that an increased pore volume fraction is expected to be accompanied by a wider distribution in pore sizes, but we observe only small differences between the two scattering curves in Figure 4a, as well as between the correlation lengths. This suggests that the scattering objects are of similar sizes and in abundance at 0 mM and 10 mM NaCl.^1^ Hence, it is more reasonable to assume that any increase in the pore volume fraction will be small.

The reduction in Δρ (eq 2) with NaCl at 100% D_2_O will be a consequence of salt-induced hydration of the pectin. Opposite to when PEMUs/PECs are formed, NaCl breaks the pairing interactions between oppositely charged biomacromolecules, and additional water molecules typically accompany the process.^32,35,36^ A D_2_O uptake in the pectin matrix will reduce the SLD contrast between the pectin phase and its surroundings. Additionally, in the fitted results (see Table S2 in the SI), we see that the SLD of the shell (core–shell sphere model) increased from 5.63 × 10^–6^ to 5.78 × 10^–6^ Å^–2^ with NaCl, which may further support increased D_2_O hydration of the shell when NaCl is present. Also, when the charge-paring interactions between pectin and d-BC are weakened, the mobility of the pectin phase is expected to increase.^1^ This can be compared with observations from other polyelectrolyte complexes, where oppositely charged polyelectrolytes can dissociate back into more mobile individual polyelectrolyte chains^38^ upon exposure to sufficiently high concentrations of salt; in other words, a PEMU/PEC can dissolve.^32^ However, the extent of this salt effect is highly dependent on the specific polyanion–polycation pair and the salt species involved.^32^ In some polyelectrolyte complexes, particularly those where the pairing is exothermic (Δ*H* < 0), the salt does not disrupt the polyanion–polycation pair but instead acts as a co-ion and stabilizes the complex^45^; however, when polycarboxylates are present, NaCl effectively breaks the pair.^32^

At 40% D_2_O, where the pectin is contrast-matched and only the d-BC contributes to the scattering signal, no significant changes were observed in the SANS curves upon NaCl addition (Figure 4b). This suggests that the d-BC nanostructure remains relatively unaffected at 10 mM NaCl. While earlier studies have reported that salt can induce aggregation or gelation in charged or uncharged nanocellulose suspensions,^46^ such effects were not detected here. The SANS scattering profile was fitted with a core–shell sphere model and the DAB model, and the results are included in the inset in Figure 4b (Table S2 in the SI). The correlation length was 71.4 Å. By doubling this (144 Å), the average spacing between regions of the same phase, here d-BC, is deduced. The fitted value is realistic, and on the same order of magnitude as the average hydrodynamic diameter (ca. 120 Å) for the 70 kDa FITC-dextran. Hence, the average distance between d-BC nanofibers within the shell is large enough to permit permeability of 70 kDa FITC-dextrans, consistent with the dextran permeability observed in the CLSM experiments (Figure 3b).

To conclude the SANS results, we show that the most pronounced structural change in the multilayer shell at 10 mM NaCl originated from the pectin phase. In biological systems, such as plant cell walls, it has been argued that pectin is the primary regulator of wall permeability; a property governed by its molecular composition and supramolecular organization.^47^

With small-angle X-ray scattering (SAXS, Figure 6a), there will only be a small difference in SLD between hydrated cellulose and hydrated pectin (14 × 10^–6^–16 × 10^–6^ Å^–2^, depending on the degree of hydration), limiting the ability to discern between the location of cellulose and pectin; however, there is a much greater difference with respect to H_2_O (SLD = 9.43 × 10^–6^ Å^–2^).^1^ The SLD difference between the water-filled pores and the surrounding hydrated d-BC/pectin matrix is approximately one order of magnitude greater.^10^ The Kratky plot in Figure 6b, calculated from the SAXS scattering data in Figure 6a, exhibited a peak at ≈0.012 Å^–1^. For comparison, the Kratky plot of previously studied wood-CNF/pectin Lbl structures in water is also shown.^1^ A shift of the peak toward lower *q*-values in the current d-BC/pectin samples is interpreted as arising from a shift to larger scattering objects (water-filled pores and core–shell structures). The full *q*-range for the Kratky plots is presented in Figure S8 in the SI.

**Figure 6:**
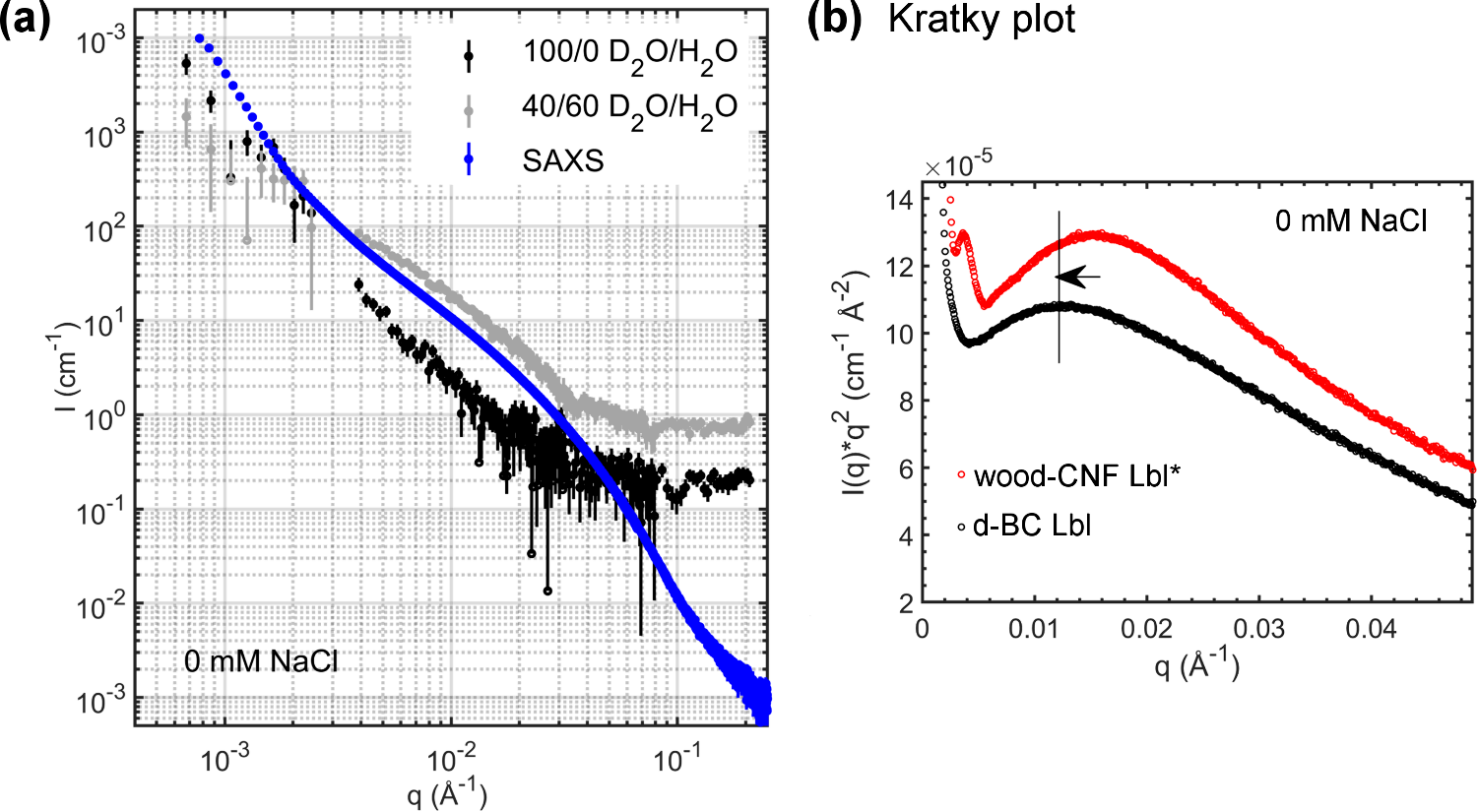
Comparison of SANS and SAXS scattering curves. (a) SAXS data for d-BC/pectin spherical core–shell structures dispersed in Milli-Q water, and SANS data for d-BC/pectin spherical core–shell structures measured in 100/0 and 40/60 D_2_O/H_2_O (without added NaCl). (b) Comparison of Kratky plots (reduced *q*-range) for d-BC/pectin and CNF/pectin core–shell structures in Milli-Q water. The Kratky plot for the d-BC/pectin sample was derived from the SAXS data in (a), and the Kratky plot for wood-CNF/pectin structures was reproduced from Mao et al.^1^ Adapted under the terms of the CC-BY 4.0 license. Copyright 2025, Elsevier.

Unlike the wood-CNF/pectin core–shell structures, the SAXS profile of the present d-BC/pectin capsules did not exhibit the characteristic shoulder at around 0.2 Å^–1^ (Figure 6a).^1^ In the previously reported wood-CNF/pectin Lbl system, this shoulder was successfully fitted using a cylinder model, where the cylinder dimensions approximately corresponded to the diameter of a wood-CNF and to the length of dislocated segments (i.e., disordered paracrystalline or “amorphous” regions) along the CNF nanofiber axis.^1,48,49^ The shoulder was therefore attributed to short disordered domains interrupting the crystalline segments of the wood-derived CNFs.^1^ The absence of a comparable shoulder in the scattering profile of the present d-BC/pectin structures can be rationalized by structural differences between bacterial cellulose and wood-derived CNFs. WAXS experiments, with results in Figure S9, confirmed that the crystalline structure of the cationically modified d-BC, incorporated into the shell of hydrated d-BC/pectin Lbl capsules, remained consistent with the cellulose Iα crystal structure typically found in bacterial cellulose.^23^

SAXS and SANS data from samples in nonsaline conditions are plotted in the same graph in Figure 6a. We note that the slope of the SANS data at 40% D_2_O (d-BC, pectin contrast-matched) was similar to that of the SAXS curve, suggesting that although the pectin and d-BC cannot be resolved separately by SAXS, the SAXS profile is dominated by the d-BC phase. This interpretation is consistent with the sugar composition analysis, which confirmed that d-BC nanofibers and crystals were the major components of the shell.

## Conclusions

We have demonstrated the utility of bioinspired core–shell structures for probing molecular architectures. Using the layer-by-layer assembly of pectin and deuterated bacterial cellulose (d-BC) nanofibers/nanocrystals on sacrificial CaCO_3_ templates, spherical capsules with controlled composition and architecture were fabricated. The resulting capsules had an average diameter of 12.7 ± 2.1 μm, a shell thickness of 280 ± 60 nm, and a cellulose-dominated composition (82.5 wt % d-BC in the dry shell). Compared to previously reported wood-CNF/pectin systems, the present d-BC/pectin shells exhibited higher permeability to 70 kDa dextran in nonsaline conditions, consistent with a more open nanofiber network and reduced pectin content. Contrast-matched SANS enabled separate characterization of pectin and d-BC networks; a 40% reduction in the scattering scale factor (*A*) was observed for the pectin phase upon addition of 10 mM NaCl, indicating increased hydration and structural loosening. Overall, these findings highlight the dominant role of pectin in mediating ionic responsiveness and wall permeability for the present synthetic systems.

This synthetic system, combined with solvent contrast variation and contrast matching through neutron scattering, provides a complementary platform for disentangling component-specific structural responses and elucidating structure–function relationships in plant cell walls. Additionally, the concept can be extended to nonplant applications and include other anionic polymers, such as alginate or hyaluronate.

## Supplementary Material



## Notes

AFM height, length, and aspect ratio distributions of cationic d-BC nanofibers/nanocrystals, diameter distribution of spherical LbL capsules, pore-size distribution of dried capsule shells, TEM cross sections and wall-thickness histogram, DLS profile of 70 kDa FITC-dextran, Kratky plots comparing d-BC/pectin and CNF/pectin capsules, contrast-matching series for pectin and d-BC, WAXS data, SANS data with core–shell sphere and DAB model fits, optimized model parameters, and monosaccharide composition of pectin and pectin recovered from capsules (PDF)
