# Supplementary material for: Disentangling Pectin and Cellulose Nanostructures in Synthetic Plant Cell Walls with Small-Angle Neutron Scattering
Source: Biomacromolecules. 2026 Apr 21;27(5):3303–13. doi: 10.1021/acs.biomac.5c02791 (PMC13169372; doi:10.1021/acs.biomac.5c02791)
Supplement: Supplementary file 1 [file bm5c02791_si_001.pdf]

SUPPORTING INFORMATION

Disentangling Pectin and Cellulose Nanostructures in Synthetic Plant Cell Walls with Small Angle Neutron Scattering

A.J. Svagan, O. Kyzyma, A. Mao, P. Sivan, H. Li, A. Ziolkowska, F. Vilaplana, R. Russell, M. Cardenas and E. P. Gilbert

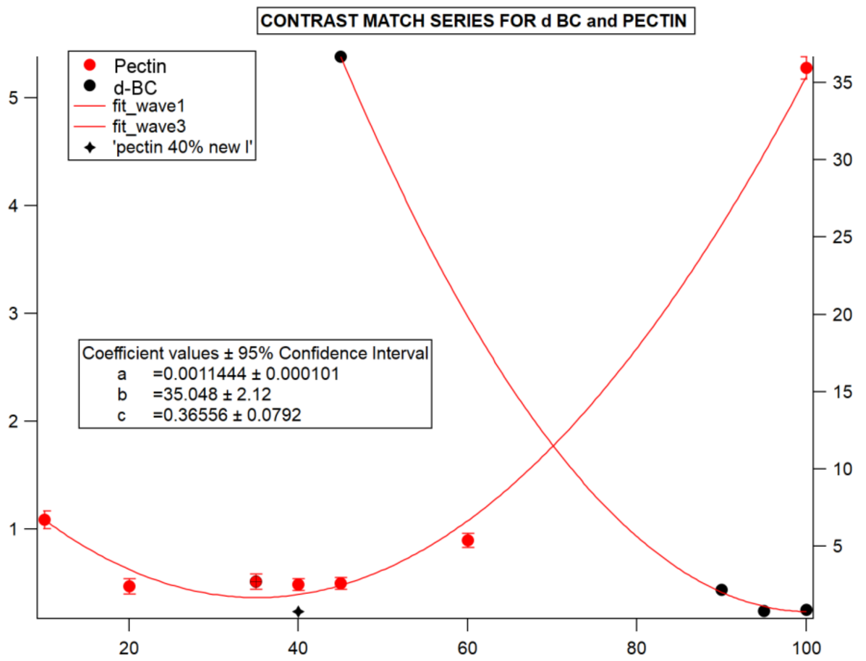

Figure S1. Solvent contrast variation data from SANS. Scattering intensity of pectin and d-BC nanofibers/nanocrystals dispersed in varying D_2O/H_2O mixtures.

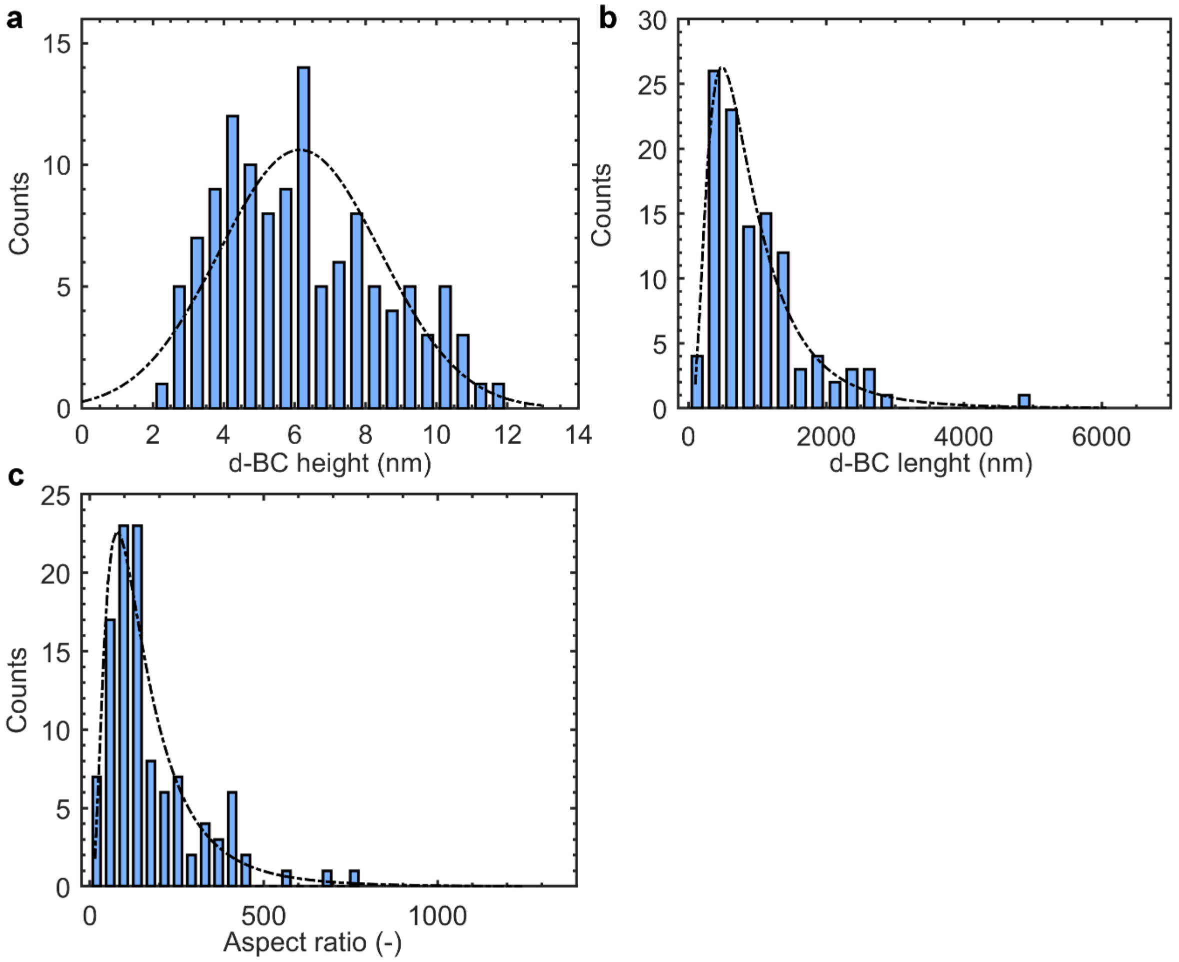

Figure S2. AFM results for cationic d-BC nanofibers/nanocrystals: (a) height (nm), (b) length (nm) and (c) Aspect ratio (= Length/height).

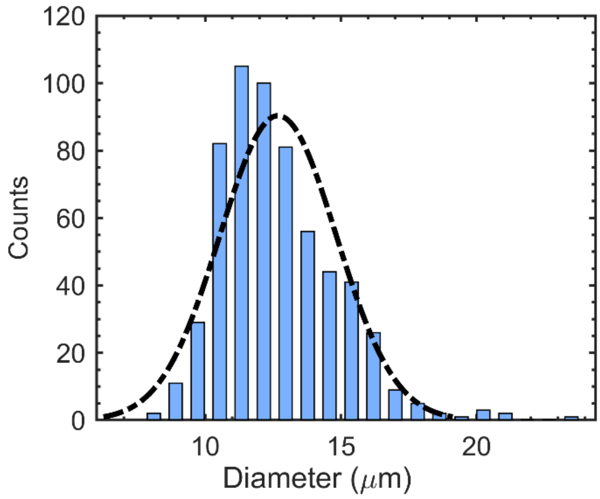

Figure S3. Histogram showing the distribution of the diameter of the Lbl capsules (n=600). The average diameter (± s.d.) is 12.7 ± 2.1 µm.

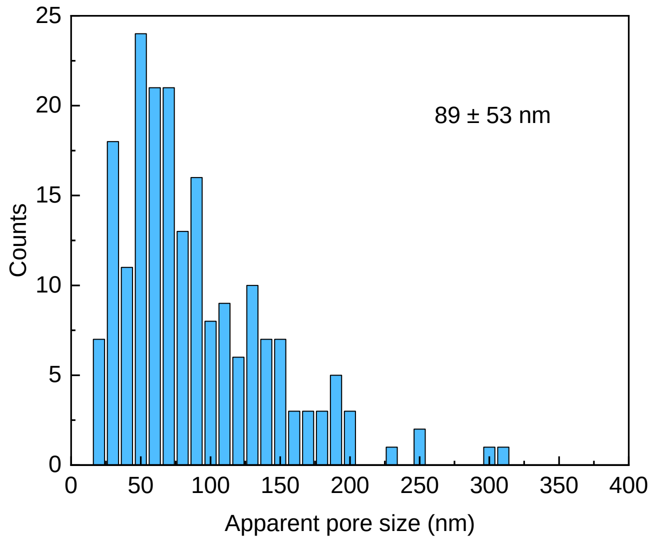

Figure S4. The diameter of voids (n= 200) identified on the surface of collapsed d-BC/pectin Lbl structures in the dry state. The average diameter is 89 ± 53 nm.

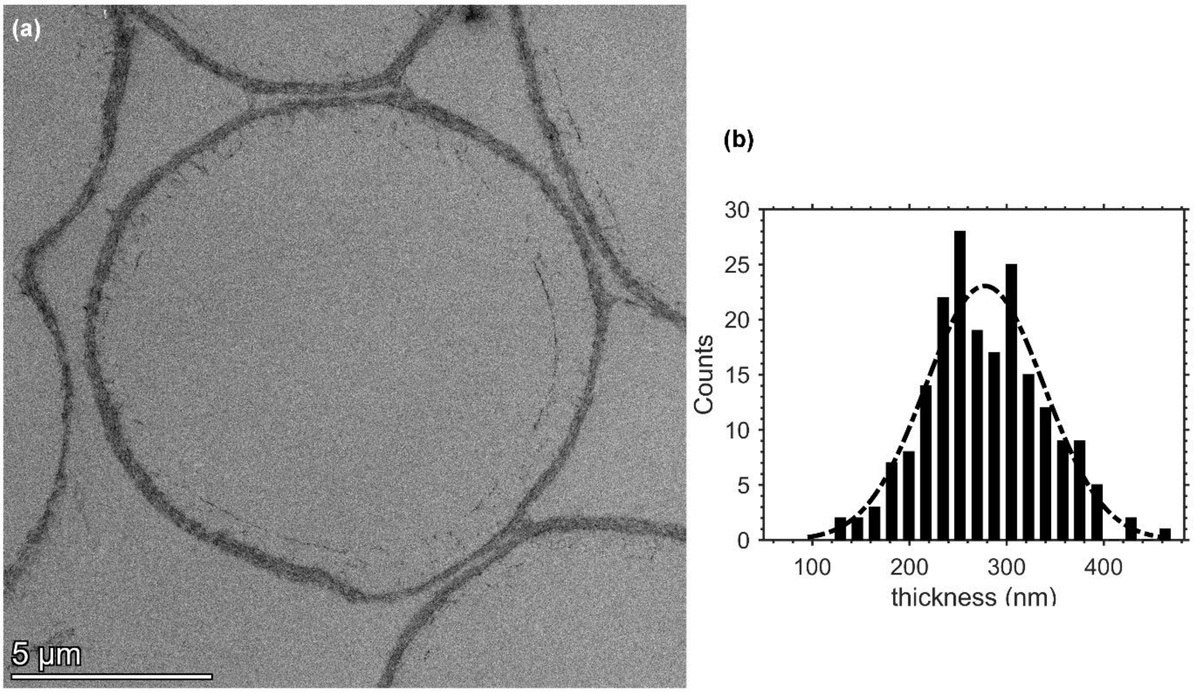

Figure S5. (a) TEM of cross-section of d-BC/pectin Lbl structures. (b) The distribution of the wall thicknesses obtained from cross-sections of d-BC/pectin structures (n=200 from 20 capsules).

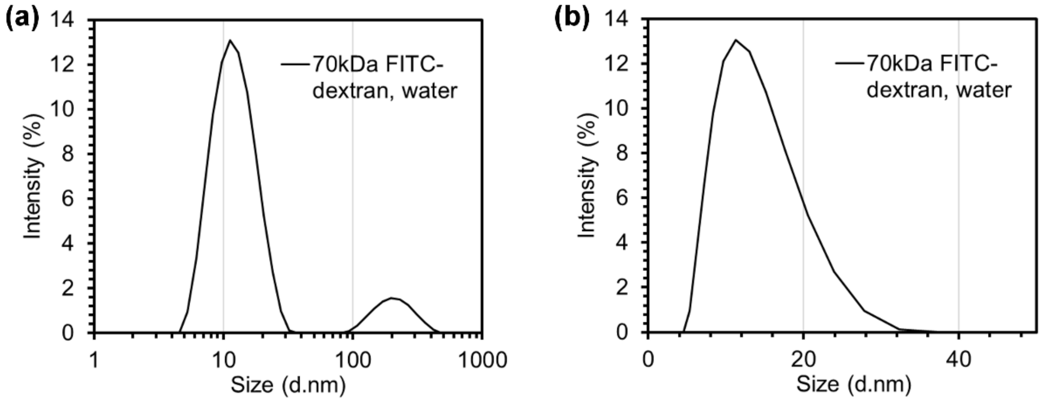

Figure S6. DLS data of 70 kDa FITC-dextran in water (0.1 wt%). The hydrodynamic diameter is presented at two different size ranges. In (b), the data are plotted on a linear scale.

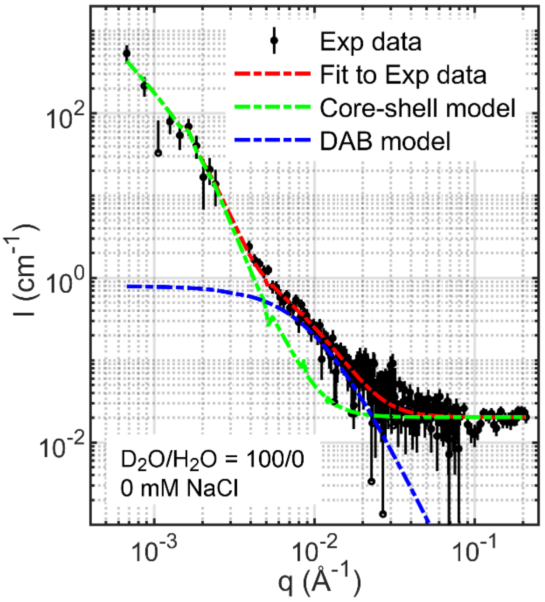

Figure S7. Experimental SANS data at D_2O/H_2O = 100/0, with 0 mM NaCl. The fit to the experimental data (dotted red line), core-shell sphere model (green) and DAB model (blue) are plotted separately.

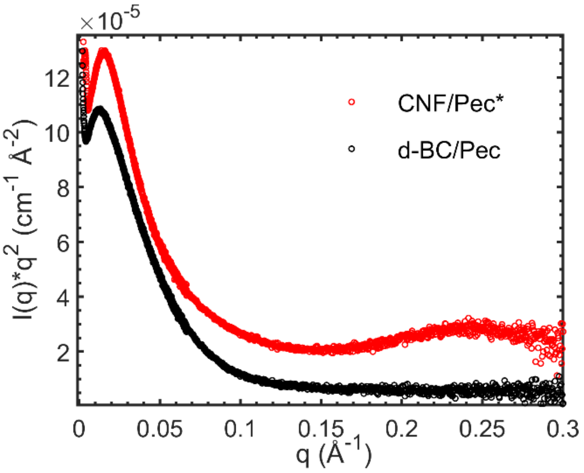

Figure S8. Kratky plots for d-BC/pectin Lbl core-shell structures in the present study (black), compared to the Kratky plot for CNF/pectin Lbl-core-shell structures from a previous study (red, data from Mao et al.[3] Adapted under the terms of the CC-BY 4.0 license. Copyright 2025, Elsevier. Both Lbl structures are present in water.

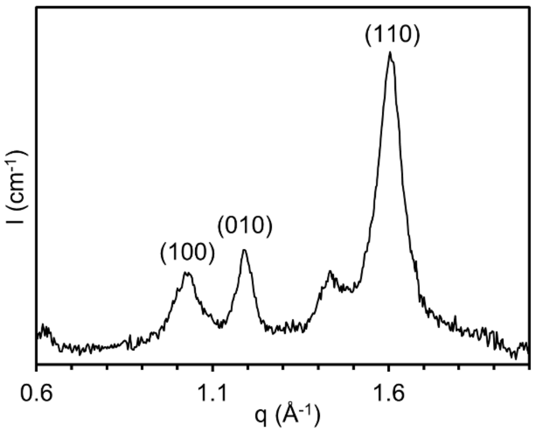

Figure S9. Wide-angle X-ray scattering (WAXS) analysis of the spherical core–shell structures. The distinct diffraction peaks ((110), (010) and (100)) correspond to the crystalline domains of the deuterated bacterial cellulose.

WAXS experiments (Fig. S9) confirmed that the crystalline structure of the cationically modified d-BC incorporated into the shell of the hydrated d-BC/pectin (Lbl) capsules (in MilliQ) remained consistent with the cellulose Iα crystal structure typically found in bacterial cellulose. The characteristic peaks at q = 1.0 $Å^{-1}$ (100), 1.2 $Å^{-1}$ (010) and 1.6 $Å^{-1}$ (110), agree with literature values.[1, 2]

Table S1. The composition of monosugars obtained from TFA hydrolysis.

Mono-sugar	Pectin (mg/g sample)	Pectin in Lbl capsules (mg/g sample)
**Fucose**	1.8	n.a.
**Rhamnose**	18.0	5.2
**Arabinose**	11.1	1.1
**Galactose**	70.3	29.4
**Glucose**	69.9	15.5
**Xylose**	13.1	4.1
**GalA**	283	39.9
**GlcA**	n.a.	7.2
**Total measured weight**	467	102

As shown in Table S1, the pectin composition in the Lbl capsules deviates from that of the starting apple pectin material, implying that specific pectin components were preferentially adsorbed during the layer-by-layer assembly.

Table S2. Optimized values attained with SasView. The values within parentheses are the uncertainties of the optimized values.

D_2O/H_2O NaCl (mM)	***100/0*** **0 mM**	**10 mM**	***40/60*** **10 mM**
	Core-shell sphere model		
**[1]Scale**	0.01 (fixed)	0.01(fixed)	0.008(fixed)
**[2]Radius of core** (Å)	63564 Å ([3])	63564 Å ([3])	63564 Å ([3])
**[2]Distribution of radius**	0.163 ([3])	0.163 ([3])	0.163 ([3])
**SLD (core, solvent,** $Å^{-2}$)	$6.36\cdot10^{-6}$ (fixed)	$6.36\cdot10^{-6}$ (fixed)	$2.208\cdot10^{-6}$ (fixed)
**SLD shell** ($Å^{-2}$)	$5.629\cdot10^{-6}$ ($\pm 0.031\cdot10^{-6}$)	$5.783\cdot10^{-6}$ ($\pm 0.033\cdot10^{-6}$)	$3.705\cdot10^{-6}$ ($\pm 0.024\cdot10^{-6}$)
**Shell thickness** (Å)	1108.9 ([3])	1109 (± 64.9)	500 (± 1.6)
**Distribution of thickness**	0.43 (± 0.10)	0.43(fixed)*	0.01 (± 0.03)
	Debye-Anderson-Brumberger (DAB) model		
**A =** $8\pi\cdot\phi(1-\phi)\Delta\rho^2$ (cm^{-1} $Å^{-3}$)	$8.0\cdot10^{-7}$ ($\pm 4.9\cdot10^{-8}$)	$4.8\cdot10^{-7}$ ($\pm 4.7\cdot10^{-8}$)	$1.1\cdot10^{-5}$ ($\pm 1.6\cdot10^{-7}$)
L = correlation length (Å)	99.8 Å (± 5.9 Å)	90.1 Å (± 8.0 Å)	72.2 Å (± 0.8 Å)
	Background		
	0.020 cm^{-1} (± 0.0004 cm^{-1})	0.017 cm^{-1} (± 0.0003 cm^{-1})	0.091 cm^{-1} (± 0.001 cm^{-1})

[1]The value is based on the approximate vol fraction of Lbl capsules. [2]The core radius and the PD for the radius is similar to that obtained with Light microscopy of d-BC/pectin Lbl capsules (PD = 0.169). The distributions (radius and shell thickness) are Gaussian, with polydispersity (PD) = std.dev/mean. [3]The input for the shell thickness, radius of core, distribution of radius was obtained from fitting SAXS data, see experimental section. *Fixed to the same value as at 0 mM NaCl.

The average radius of the Lbl capsules, which includes the water-filled core and the shell, was obtained with Light microscopy and was 63400 Å in water (diameter 12.7 ± 2.1 µm). Also, we note we have limited sensitivity to dimensions of the larger core-shell sphere lengths, because the lowest q value in the experiment is 0.0007 $Å^{-1}$, hence the shell thicknesses reported in Table S2 should be seen as approximate values.

REFERENCES

1. Martínez-Sanz, M.; Mikkelsen, D.; Flanagan, B.; Gidley, M. J.; Gilbert, E. P., Multi-scale model for the hierarchical architecture of native cellulose hydrogels. *Carbohydrate Polymers* **2016,** *147*, 542-555.
2. Mehta, K.; Pfeffer, S.; Brown, R. M., Characterization of an acsD disruption mutant provides additional evidence for the hierarchical cell-directed self-assembly of cellulose in Gluconacetobacter xylinus. *Cellulose* **2015,** *22* (1), 119-137.
3. Mao, A.; Ziolkowska, A.; Paulraj, T.; Riazanova, A.; Kyzyma, O.; Gilbert, E. P.; Cardenas, M.; Svagan, A. J., Assessing porosity in cellulose nanofiber-based artificial plant cell walls as a function of salinity. *Carbohydrate Polymers* **2025**, 124296.
